# Low graft failure and favourable outcomes after anterior cruciate ligament reconstruction and lateral extra‐articular tenodesis in young athletes

**DOI:** 10.1002/jeo2.70524

**Published:** 2025-11-16

**Authors:** Mihail Lazar Mioc, Blaithin Brady, Anna Rose O'Brien, Mihai Vioreanu

**Affiliations:** ^1^ UPMC Sport Surgery Clinic Dublin Ireland; ^2^ Department XV Orthopedics and Traumatology Victor Babes University of Medicine and Pharmacy Timisoara Romania

**Keywords:** anterior cruciate ligament reconstruction, graft survivability, lateral extra‐articular tenodesis, return to sport, young athletes

## Abstract

**Purpose:**

Young athletes undergoing anterior cruciate ligament reconstruction (ACLR) are at high risk of graft failure and contralateral anterior cruciate ligament (CACL) injury, despite advances in surgical technique. Lateral extra‐articular tenodesis (LET) has emerged as a potential adjunct to improve graft survivability in high‐risk young populations. The study aimed to describe graft survival, CACL rates, patient‐reported outcome measures (PROMs) and return‐to‐sport (RTS) outcomes in high‐risk under‐20 athletes undergoing ACLR and LET.

**Methods:**

This retrospective cohort study analysed U20 athletes who underwent primary ACLR with hamstring tendon autografts and concurrent LET between 2017 and 2023. All surgeries were performed by a single surgeon using a standardized technique. Outcomes included graft survivability, CACL injury incidence, and RTS level, with functional recovery assessed via the Marx Activity Scale and Tegner–Lysholm Score. Kaplan–Meier survival analysis, independent *t*‐tests, *χ* ^2^ tests, and multivariate logistic regression were used to evaluate outcomes and identify predictors, with significance set at *p* < 0.05.

**Results:**

One hundred fifty‐nine patients (mean age 17.6 ± 1.9 years) were included, with a mean follow‐up of 49.7 ± 18.8 months. Graft rerupture occurred in 2.5% of patients, all within 40 months postoperatively. CACL injury was observed in 12.6% of patients. Over 60% of athletes returned to their pre‐injury level of sport, with higher PROMs in those achieving same‐level RTS. A smaller graft diameter was significantly associated with increased risk of CACL injury (*p* < 0.05). LET was not associated with adverse effects on functional recovery.

**Conclusions:**

This cohort showed low graft rerupture rates, favourable functional outcomes and encouraging RTS levels following ACLR and LET. However, CACL injury remained a substantial concern. LET may be considered in adolescent athletes participating in pivoting sports to potentially reduce graft failure, although further comparative studies are needed.

**Level of Evidence:**

Level IV.

## Graphical abstract

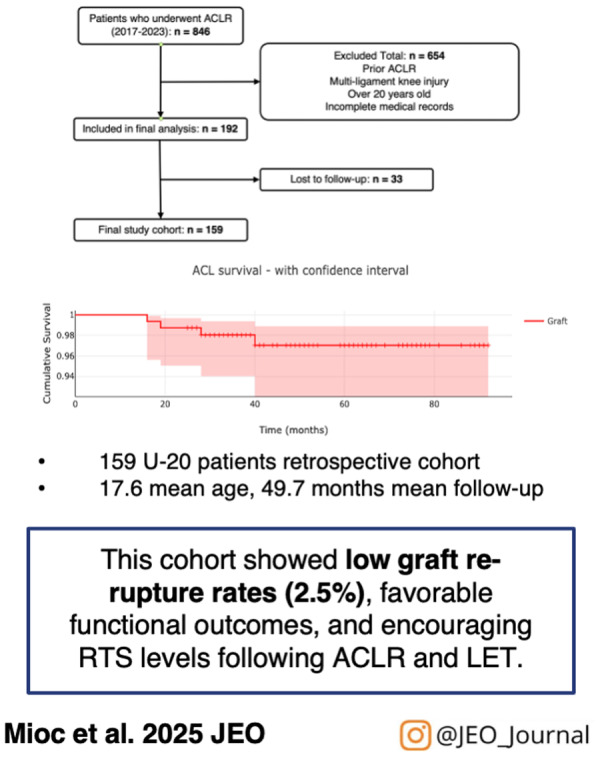

**Abbreviations**
ACL: anterior cruciate ligament
ACLR: anterior cruciate ligament reconstruction
CACL: contralateral anterior cruciate ligament
CI: confidence interval
HT: Hamstring tendons
IQR: interquartile range
ITB: iliotibial band
LCL: lateral collateral ligament
LET: lateral extra‐articular tenodesis
OR: odds ratio
PROMs: patient‐reported outcome measures
RTS: return to sport
SD: standard deviation
STROBE: strengthening the reporting of observational studies

## INTRODUCTION

The anterior cruciate ligament (ACL) is essential for knee stability, especially during high‐demand sports that involve dynamic movements and rapid changes in direction. ACL injuries are among the most common and severe sports‐related knee injuries, often leading to significant time away from sport, psychological distress and a long rehabilitation process [5, 26]. Certain sports activities, such as rugby, football, gaelic football and hurling, involve frequent pivoting, cutting and rapid directional changes, significantly stressing the ACL [7, 19, 25]. After anterior cruciate ligament reconstruction (ACLR), many of these athletes aim to return to their preinjury level of sport, a goal that inherently places them at an increased risk for graft rerupture and contralateral anterior cruciate ligament (CACL) injury [23]. The challenge of balancing early return to sport (RTS) with long‐term knee health remains a central issue in managing this young population.

Despite advances in surgical technique and rehabilitation, failure rates in this population can reach rates of 25% [3, 22, 34]. Hamstring tendon (HT) autografts, a common choice for ACLR in this young population, are associated with higher rerupture rates [11, 27]. This likely reflects a combination of biological immaturity, high activity levels, early RTS and technical factors, making this group especially vulnerable. Furthermore, recent studies have shown that patients in this cohort had an increased risk of a subsequent ACL injury, regardless of the graft type, mostly between 2 and 5 years after reconstruction [15].

Lateral extra‐articular tenodesis (LET) has recently gained attention as an adjunct to primary ACLR, because it increases rotational stability and may reduce graft failure in high‐risk athletes [2, 6, 10, 14, 20, 29]. Despite that, data specifically focusing on homogenous under‐20 cohorts with standardized surgical technique and rehabilitation protocols remain limited. Moreover, few studies report mid‐term functional outcomes and RTS levels in this population. Addressing these gaps may provide clinicians with more reliable information when counselling high‐risk adolescent athletes and their families.

The study aims to describe graft survival, CACL rates, PROMs and return‐to‐sport (RTS) outcomes in high‐risk under‐20 athletes undergoing ACLR and LET. Our hypothesis is that performing an LET will positively influence graft survivability in this patient cohort, improving RTS outcomes and mid to long‐term functional recovery.

## MATERIALS AND METHODS

### Study design

This is a retrospective analysis of a prospectively followed cohort of U20 athletes who underwent ACLR with additional LET between March 2017 and March 2023. The study describes graft survivability, CACL injury rates, RTS outcomes and patient‐reported outcome measures (PROMs). All procedures were performed by a single surgeon using a standardized technique. Outcomes were assessed at follow‐up periods ranging from 2 to 8 years. This study was conceived adhering to the STROBE (Strengthening the Reporting of Observational Studies in Epidemiology) guidelines to ensure transparent and accurate reporting of observational research [31]. The STROBE checklist was used to structure the study design, data collection, analysis and reporting. This study adheres to the Declaration of Helsinki and approval from our institution's ethics committee was obtained per reference number MiVi_SSC_2021_ACLR. Informed consent for data collection and publication was obtained from all patients or their legal guardians.

### Patient selection

Consecutive patients were included in our analysis based on certain inclusion and exclusion criteria. The inclusion criteria were:

1. Primary traumatic ACL rupture requiring ACLR and LET.
2. Age under 20 years at the time of surgery.
3. Active participation in high to very high‐intensity sports activities.
4. Availability for follow‐up of at least 2 years.

The exclusion criteria were:

1. The existence of multiligament knee injuries.
2. History of prior ACLR or CACL reconstruction.
3. Incomplete medical records (e.g., missing operative details or rehabilitation notes).
4. Inability to contact for follow‐up despite at least three phone attempts.

This selection ensured a homogenous cohort of young, high‐risk athletes engaging in competitive, high‐contact sports requiring increased biomechanical demands such as cutting, pivoting and jumping. The study size was determined by the total number of consecutive patients meeting the inclusion criteria in a set time interval. No formal power analysis was performed, as this was a retrospective cohort based on all eligible cases within the study period. To reduce selection bias, all consecutive patients over the study period were included. Data were collected from standardized operative reports and follow‐up protocols, minimizing information bias. Outcome assessments were based on validated PROMs administered by trained staff via structured phone interviews.

### Surgical technique and rehabilitation

All ACLRs in this study were performed using quadruple HT autografts exclusively, in one single centre by a single surgeon. All procedures were performed under general anaesthesia, under antibiotic prophylaxis, with the tourniquet positioned as proximally as possible on the thigh and with the knee in extension (no leg‐holder). The tibial insertion was kept for both tendons upon stripper harvesting (Linvatec, ConMed Corporation) and detached just before graft‐tunnel passage; these were quadrupled over 2× #5 Fiberwire sutures (Arthrex) used for graft‐tunnel passage and whip‐stitched using one #2 Vicryl suture (Ethicon) at each end. All grafts were presoaked in a vancomycin solution until the ACL remnant had been debrided, and the tunnels had been drilled.

The femoral tunnel was drilled through the anteromedial portal by overdrilling with a stepped router over a 4.5 mm previously drilled hole. This allowed for a 30 mm deep femoral socket on the native ACL footprint. The tibial tunnel was drilled in a standard fashion with a tibial drilling guide (Smith & Nephew) set at 55°, again with a 4.5 mm drill followed by a stepped router that created a 45 mm tibial socket. Aperture femoral and tibial fixation was performed with the aid of two polyether ether ketone (PEEK) screws (RCI, Signature Orthopedics). Tibial fixation was performed after the LET procedure, with the knee in extension/hyperextension. The LET was performed in all patients using the modified Lemaire technique [33]. This was achieved by passing a strip of the iliotibial band deep and proximal to the lateral collateral ligament (LCL) and fixing it on the lateral femoral condyle with an all‐suture anchor.

All patients were advised to follow the same accelerated rehabilitation protocol designed by the main surgeon and his team, which was given to them in written format. No knee braces were used, and patients were fully weight‐bearing immediately after surgery. This protocol included specific postoperative periods that ended with clinical follow‐ups (6 days, 6 weeks and 6 months) performed by the surgical team. This protocol was designed to allow for RTS around the 12‐month period.

### Outcome measures and data collection

The primary outcome measures of this study include graft survivability and the incidence of CACL injuries. Graft failure was defined as a confirmed rerupture of the reconstructed ACL, diagnosed by clinical examination (positive Lachman and/or pivot shift consistent with instability) and magnetic resonance imaging (MRI) findings, and in all cases confirmed by the need for revision ACLR. CACL injury was considered present if the patient sustained an ACL rupture in the contralateral knee during the follow‐up period.

Secondary outcomes measured functional recovery and RTS levels using validated PROMs. All patients were contacted by phone over a time span of 6 weeks and asked to complete the Marx Activity Scale and Tegner–Lysholm Score, both validated instruments for evaluating activity and knee function in young athletic populations [13, 30]. As all study participants were native English speakers, the original validated versions of these PROMs did not require any translation. RTS levels were also assessed subjectively during the phone call. Those who could not be reached after three separate attempts over this time were classified as lost to follow‐up. No substitution was performed for missing outcome data. As young, high‐level sports practitioners, they were presumed to have maximal baseline scores on both scales. Demographic data, including gender and age at surgery, were taken from medical records. In addition, intraoperative data were reviewed to document the presence of medial and/or lateral meniscal lesions, whether or not these were repaired, and the diameter of the ACL graft used in each case.

### Statistical analysis

All statistical analyses were conducted using Microsoft Excel® (Microsoft Corporation) and JASP® (Version 0.19.3, JASP Team). Descriptive analysis, including means, standard deviations and frequencies, was calculated in Excel to summarize baseline patient characteristics. Normality of continuous variables was assessed using the Shapiro–Wilk test. Comparative analyses between groups were performed in JASP: independent *t*‐tests were used to compare continuous variables, such as PROMs, while nonparametric tests were applied when normality assumptions were not met. *χ* ^2^ tests were used for categorical variables, such as rerupture incidence and CACL injuries. Kaplan–Meier survival analysis was conducted in JASP to evaluate time to ACL graft failure and CACL injuries over the follow‐up period. Survival curves were compared using the log‐rank test to determine the statistical significance between subgroups. A multivariate logistic regression model was performed to identify independent predictors of RTS, ACL graft survivability, and CACL injury risk, adjusting for age and gender. A post hoc power analysis was performed to compare the observed graft rate with a 20% benchmark failure rate reported in the literature. No sensitivity analyses were performed due to the limited number of outcome events and the uniform cohort design. Statistical significance was set at *p* < 0.05, and all confidence intervals (CIs) were reported at the 95% level.

## RESULTS

### Patient demographics

**Figure 1:**
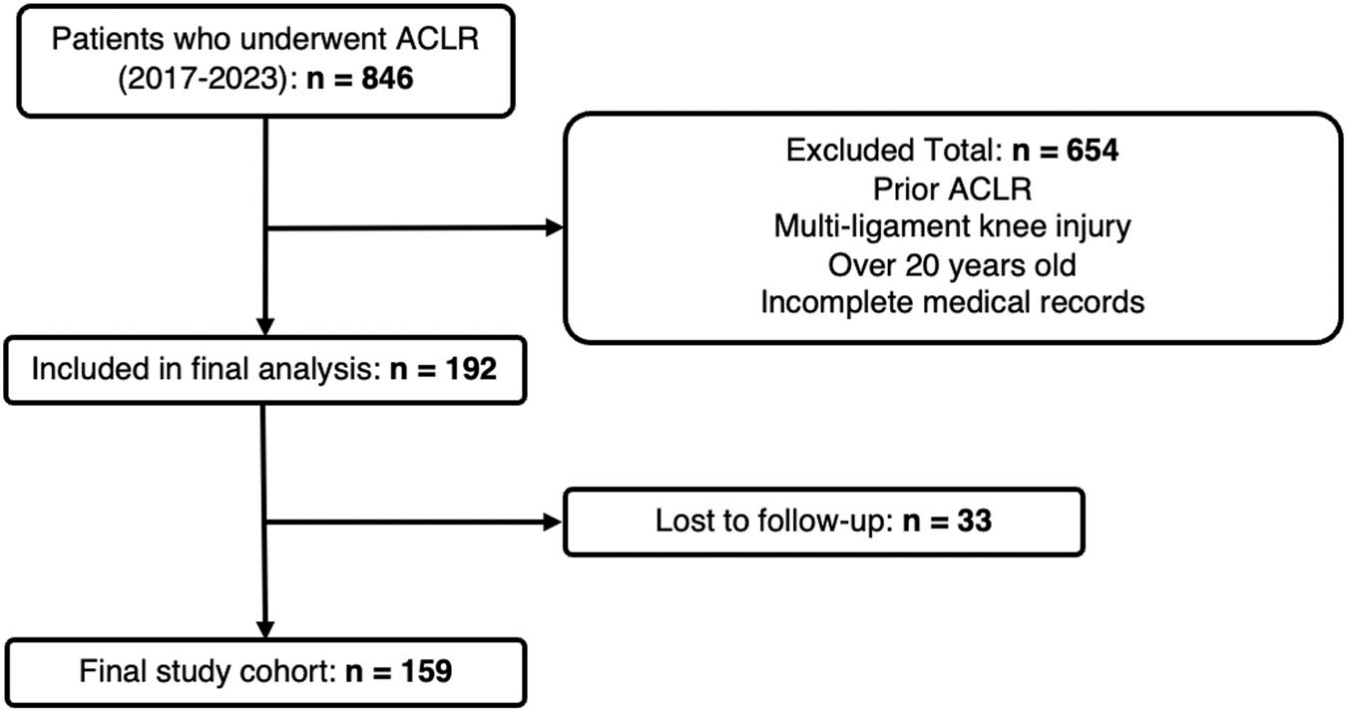
Study cohort selection and exclusion pathway (adapted from CONSORT). ACLR, anterior cruciate ligament reconstruction.

Of the 846 ACLRs performed during the study period, 654 were excluded due to not meeting the inclusion criteria: 544 were older than 20 years at surgery, 81 were revision ACLRs, 17 were multi‐ligament injuries and 12 had incomplete records. The patient inclusion flowchart is shown in Figure 1.

Out of an initial cohort of 192 patients (after inclusion and exclusion criteria), 33 (17.2%) were lost to follow‐up, resulting in 159 patients included in the final analysis. The cohort consisted of 94 males (59.1%) and 65 females (40.9%), with a mean age at surgery of 17.6 ± 1.93 years, with male patients being slightly older than females (18 ± 1.90 vs. 17.1 ± 1.86 years). The mean follow‐up was 49.7 ± 18.8 months with a median follow‐up of 47 months (interquartile range [IQR]: 32). All patient baseline characteristics can be found in Table 1.

**Table 1:** Baseline clinical and surgical characteristics of the study cohort.

Characteristic	Male	Female	Overall
Age at surgery (years)	18.0 ± 1.90 (13–20)	17.1 ± 1.86 (14–20)	17.6 ± 1.93 (13–20)
Follow‐up duration (months)	50.1 ± 19.6 (25–92)	49.2 ± 17.9 (25–90)	49.7 ± 18.83 (25–92)
Medial meniscal lesions, *n* (%)	40 (42.6%)	25 (38.5%)	65 (40.9%)
Medial meniscal repairs, *n* (% of lesions)	32 (80%)	23 (92%)	55 (84.6%)
Lateral meniscal lesions, *n* (%)	55 (58.5%)	38 (58.5%)	93 (58.5%)
Lateral meniscal repairs, *n* (% of lesions)	44 (80%)	32 (84.2%)	76 (81.7%)
Graft diameter (mm)	7.78 ± 0.53 (6.5–8.5)	7.28 ± 0.52 (6.5–8.5)	7.57 ± 0.58 (6.5–8.5)

*Note*: Descriptive statistics for patient demographics and intraoperative findings. Values are reported as mean ± standard deviation (SD) with range or as absolute numbers with percentages. Meniscal repair rates are presented as the percentage of repairs performed among patients with confirmed lesions.

### Graft survival

**Figure 2:**
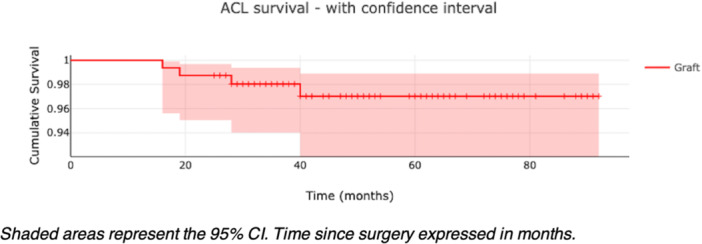
Kaplan–Meier curve illustrating ACL graft survivability. Shaded areas represent the 95% CI. Time since surgery expressed in months. ACL, anterior cruciate ligament; CI, confidence interval.

**Figure 3:**
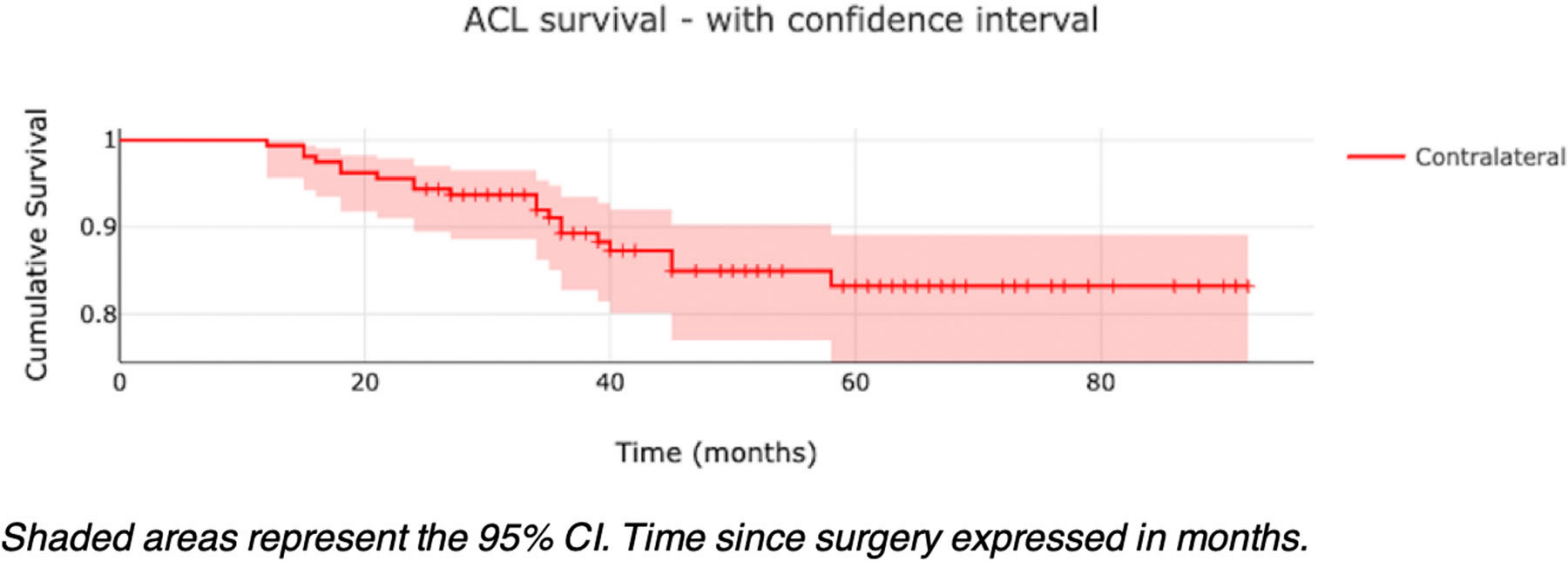
Kaplan–Meier curve illustrating CACL survivability. Shaded areas represent the 95% CI. Time since surgery expressed in months. CACL, contralateral anterior cruciate ligament; CI, confidence interval.

Graft rerupture occurred in 4 patients (2.5%) and contralateral ACL rupture in 20 patients (12.6%). Kaplan–Meier curves illustrated graft and CACL survival (Figures 2 and 3), with time‐stratified breakdowns shown in Tables 2 and 3. A post hoc power analysis confirmed that the study was well‐powered to detect clinically meaningful differences, compared to the 20% benchmark failure rate. With an alpha of 0.05, a sample size of 159 patients and 4 events, the observed power was 99.97%, indicating that the study was adequately powered to detect a clinically meaningful difference. No gender‐based differences in survival outcomes were observed, and meniscal lesion status at the time of surgery was not associated with graft or CACL survival.

**Table 2:** Graft rerupture events stratified by time since surgery.

Time interval	Total patients	Graft reruptures (*n*)	Rerupture times (months)	Censored (*n*)	Rerupture rate (95% CI)
<36 months	49	3	16, 19, 28	46	6.1% (2.1%–16.5%)
36–60 months	55	1	40	54	1.8% (0.3%–9.6%)
>60 months	55	0	—	55	0.0% (0.0%–6.8%)

*Note*: Distribution of graft rerupture events grouped by time since index ACL reconstruction with LET. Time intervals reflect the moment of the event for patients with graft failure and total follow‐up duration for censored cases. The table includes the number of reruptures, exact timing of events, number of censored patients and 95% CIs for the event rate within each time window

Abbreviations: ACL, anterior cruciate ligament; CIs, confidence intervals; LET, lateral extra‐articular tenodesis.

**Table 3:** CACL rupture events stratified by time since surgery.

Time interval	Total patients	CACL ruptures (*n*)	Rupture times (months)	Censored (*n*)	Rupture rate (95% CI)
<36 months	49	8	15, 17, 18, 19, 20, 26, 30, 35	41	16.3% (8.6%–28.7%)
36–60 months	55	11	36, 38, 39, 40, 42, 43, 44, 45, 48, 48, 55	44	20% (11.7%–32.26%)
>60 months	55	1	63	54	1.8% (0.3%–9.6%)

*Note*: Distribution of CACL rupture events grouped by time since index ACL reconstruction with LET. Time intervals reflect the moment of the event for patients with CACL failure and total follow‐up duration for censored cases. The table includes the number of CACL ruptures, exact timing of events, number of censored patients and 95% CIs for the event rate within each time window

Abbreviations: ACL, anterior cruciate ligament; CACL, contralateral anterior cruciate ligament; CIs, confidence intervals; LET, lateral extra‐articular tenodesis.

### Functional outcomes and RTS

**Figure 4:**
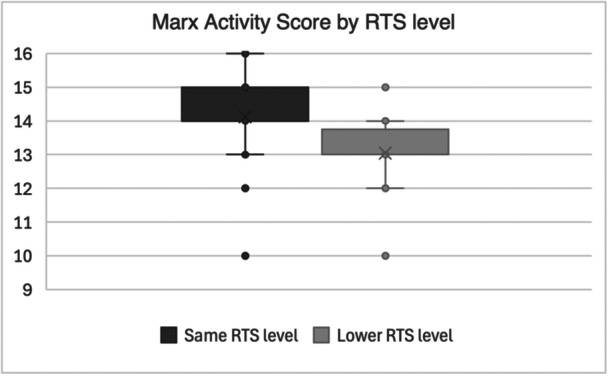
Boxplot illustrating Marx Activity Scale scores by RTS levels. The boxes represent the IQR, the horizontal line indicates the median, the ‘x’ marks the mean, and the whiskers denote minimum and maximum values with the 1.5 IQR. Individual dots represent outliers. IQR, interquartile range; RTS, return to sport.

**Figure 5:**
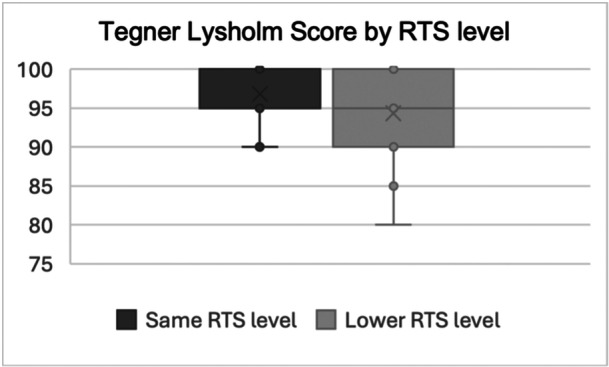
Boxplot illustrating Tegner–Lysholm Scores by RTS levels. The boxes represent the IQR, the horizontal line indicates the median, the ‘x’ marks the mean, and the whiskers denote minimum and maximum values with the 1.5 IQR. Individual dots represent outliers. IQR, interquartile range; RTS, return to sport.

The overall functional outcomes of the cohort were high, with mean Marx and Tegner–Lysholm Scores indicating good activity levels and knee function (Table 4). A ceiling effect was noted in both scores. Overall, 62% of patients returned to their preinjury sport level, while 38% returned at a lower level. Patients achieving same‐level RTS reported significantly higher PROMs (Figures 4 and 5).

**Table 4:** Summary of clinical outcomes.

Outcome	Males (*n* = 94)	Females (*n* = 65)	Overall (*n* = 159)	*p* value
Same level RTS, *n* (%)	59 (62.8%)	40 (61.5%)	99 (62.3%)	0.996
Lower level RTS, *n* (%)	35 (37.2%)	25 (38.5%)	60 (37.7%)	0.977
Graft rerupture, *n* (%)	3 (3.2%)	1 (1.5%)	4 (2.5%)	n/r
Mean time to graft rerupture (months)	31.7 ± 12.7	36 (single case)	33.0 ± 14.5	n/r
CACL rupture, *n* (%)	12 (12.8%)	8 (12.3%)	20 (12.6%)	0.258
Mean time to CACL rupture (months)	28.6 ± 16.3	28 ± 14.5	28.3 ± 15.6	n/r
Marx Activity Score (mean ± SD)	13.6 ± 1.7	13.4 ± 1.9	13.5 ± 1.8	0.075
Tegner–Lysholm Score (mean ± SD)	94.5 ± 6.4	94.9 ± 5.8	94.6 ± 6.1	0.193

*Note*: Graft rerupture and contralateral ACL rupture were recorded as binary outcomes. Time to event values are expressed in months. *p* values represent comparisons between male and female patients using *χ* ^2^ or independent *t*‐tests, as appropriate.

Abbreviations: CACL, contralateral anterior cruciate ligament; n/r, not reported; RTS, return to sport; SD, standard deviation.

### Postoperative complications

Early postoperative complications occurred in 22 patients (13.8%). Most were minor and transient, including 16 cases of temporary extension lag and 5 haematomas at the LET site, all resolving with conservative treatment. One patient required minor revision for superficial wound dehiscence. No deep infections, thromboembolic events or hardware‐related issues were observed.

### Predictors of outcomes

Age and gender were not associated with graft rerupture, CACL injury or RTS level. A smaller graft diameter was significantly linked to higher CACL injury risk. In multivariate logistic regression including age, gender and meniscal status, only repaired lateral meniscal lesions predicted a greater likelihood of the same level RTS (odds ratio [OR]: 2.07, *p* = 0.04). Similarly, no patient or intraoperative factors were significantly associated with either the Marx Activity Scale or the Tegner–Lysholm Score.

## DISCUSSION

This study evaluated the mid‐term outcomes of combining ACLR with LET in high‐risk athletes under 20 years old. Our findings revealed low graft failure rates, consistent with prior literature suggesting that LET may reduce rerupture risk in high‐risk populations. LET did not appear to influence CACL rupture rate, which remained a substantial risk in this cohort.

### Graft rerupture rate

In this prospective cohort of U20 athletes undergoing HT ACL reconstruction combined with LET, the observed graft rerupture rate was notably low, at 2.5% over a median follow‐up of nearly 4 years. This rate is notably low when compared with graft failure rates of 10%–25% previously reported in similar high‐risk populations that did not undergo LET [3, 6]. Although our study lacks a control group, these findings suggest that the use of LET may be associated with favourable graft survival in young athletes participating in pivoting sports. These findings align with the current literature, suggesting that the addition of a lateral stabilization procedure, whether LET or anterolateral ligament (ALL) reconstruction, indeed reduces ACL graft failure in this population group. The STABILITY study showed a 67% relative risk reduction in graft rerupture among young, elite athletes when LET was added to their ACL reconstruction [8]. Similarly, Sonnery‐Cottet et al. reported superior graft survival (2.5 times less than BTB and 3.1 times less than HT without ALL) and lower revision rates with combined intra‐ and extra‐articular reconstruction techniques compared to isolated ACLR [28, 29]. While our study differs in design and population, the 2.5% rerupture rate observed in our cohort—all of whom received LET—is substantially lower than historical rerupture rates of to 25% in this demographic when LET is not performed.

Our data also showed that all reruptures occurred within the first 40 months postsurgery, with no events recorded in patients followed beyond 5 years. This pattern may reflect the well‐documented increased vulnerability of the graft during the early RTS window, when biological graft maturation and neuromuscular adaptations are still incomplete [21, 22]. While the absence of failures after 3.5 years is encouraging, it may also reflect decreased sporting intensity as patients age. As our cohort lacked a non‐LET comparator, further prospective studies are needed to determine whether LET extends long‐term graft protection or primarily mitigates early failure.

### CACL injury

In contrast to the low graft failure rate, the incidence of CACL injury in our cohort was 12.6%, consistent with rates reported in young athletic populations [9, 18, 24, 32]. This underscores that LET, while improving ipsilateral stability, does not protect the contralateral limb. CACL injuries often reflect a complex interplay of biomechanical risk factors, neuromuscular deficits, collagen issues, behavioural patterns, as well as RTS exposure. A higher CACL rupture rate may paradoxically indicate successful return to high‐intensity sport, whereas lower rates could reflect reduced participation due to poor recovery or graft failure. The significant association between smaller graft diameter and CACL injury suggests an intrinsic predisposition in some patients, warranting future investigation. These findings highlight the need for bilateral neuromuscular prevention strategies in this population.

### RTS and PROMs

RTS outcomes in this cohort were encouraging, with over 60% of patients returning to their preinjury level of participation. This aligns with findings from systematic reviews, which reported that approximately 63% of individuals returned to their preinjury level of sport following ACLR [1]. More recently, a systematic review published in 2024, focusing specifically on soccer players, found that 72% of athletes return to play, but only 53% achieve RTS at their preinjury level, with a mean return time of 8.7 months [12]. Migliorini et al. reported that approximately 72% of athletes who underwent ACLR and LET returned to their preinjury level of sport [16]. Supporting this further, a systematic review performed on skeletally immature patients undergoing combined ACLR and LET, reporting favourable outcomes of 95% overall RTS [4].

Patients in our study who returned to the same level of sport reported significantly higher Marx Activity and Tegner–Lysholm Scores, reinforcing the association between subjective functional recovery and return to performance. This relationship has been variably observed across the literature; for instance, a systematic review highlighted that while PROMs often improve after ACLR, they do not always align with successful return to preinjury performance, particularly in elite athletes [17]. In contrast, our findings suggest a more consistent link between perceived function and actual RTS. The high PROMs observed across the cohort, with minimal ceiling effect, suggest that combining ACLR with LET does not impair, and in fact may support functional outcomes. LET may contribute to both mechanical stability and subjective confidence—critical factors for athletes aiming to resume high‐demand sports. From a clinical standpoint, these findings support the routine consideration of LET in high‐risk adolescents and young adults undergoing ACLR, particularly in those engaging in pivot‐heavy sports like gaelic football, rugby and soccer.

In our regression analysis, baseline demographic factors such as age, gender and graft diameter did not show significant influence on functional outcomes. The explanatory power of these models was negligible, suggesting that PROMs in this cohort are likely driven by other variables not captured in our dataset. This can potentially be linked to rehabilitation quality, psychological readiness or sport‐specific demands.

### Strengths and limitations

Although the protective role of LET on graft failure is increasingly recognized, our study contributes novelty by reporting one of the largest U20 athlete cohorts with consistent surgical technique and extended follow‐up. The focus on a high‐risk, homogenous athletic population enhances the relevance of our findings, particularly in sports requiring frequent pivoting and cutting. Additionally, the use of both objective surgical data and validated PROMs allows for a multifaceted evaluation of graft survivability, functional recovery and RTS.

However, several limitations must be acknowledged. First, while this is a retrospective analysis, the cohort was prospectively followed, which improves data integrity but still introduces potential limitations in study design and confounder control. Secondly, the absence of a control group without LET limits our ability to directly attribute observed outcomes to the LET procedure itself. Third, while follow‐up was robust, RTS level was self‐reported and not externally validated, potentially introducing subjectivity and recall bias. Finally, the low number of graft re‐ruptures limited our ability to perform subgroup analyses or identify specific predictors of failure. These limitations should be addressed in future prospective, controlled studies to refine patient selection criteria and optimize surgical strategies in young, high‐risk athletes.

## CONCLUSION

This cohort showed low graft rerupture rates, favourable functional outcomes and encouraging RTS levels following ACLR and LET. However, CACL injury remained a substantial concern. LET may be considered in adolescent athletes participating in pivoting sports to potentially reduce graft failure, although further comparative studies are needed.

## AUTHOR CONTRIBUTIONS

**Mihail Lazar Mioc**: Acquisition; analysis; writing; review. **Blaithin Brady**: Acquisition; investigation; supervision. **Anna Rose O'Brien**: Acquisition; investigation; supervision. **Mihai Vioreanu**: Conceptualization; main surgeon; methodology supervision; review.

## CONFLICT OF INTEREST STATEMENT

The authors declare no conflicts of interest.

## ETHICS STATEMENT

Approval obtained per reference number MiVi_SSC_2021_ACLR. Explicit consent to participate was not required, as retrospective chart reviews are considered low‐risk studies under institutional guidelines.

## Data Availability

Data are available on request from the authors.
